# Two Lysin-Motif Receptor Kinases, Gh-LYK1 and Gh-LYK2, Contribute to Resistance against *Verticillium wilt* in Upland Cotton

**DOI:** 10.3389/fpls.2017.02133

**Published:** 2017-12-13

**Authors:** Zhouhang Gu, Tingli Liu, Bo Ding, Fangfang Li, Qian Wang, Shasha Qian, Fei Ye, Tianzi Chen, Yuwen Yang, Jinyan Wang, Guoliang Wang, Baolong Zhang, Xueping Zhou

**Affiliations:** ^1^State Key Laboratory of Rice Biology, Institute of Biotechnology, Zhejiang University, Hangzhou, China; ^2^Zhejiang Province Key Laboratory of Plant Secondary Metabolism and Regulation, College of Life Science, Zhejiang Sci-Tech University, Hangzhou, China; ^3^Provincial Key Laboratory of Agrobiology, Jiangsu Academy of Agricultural Sciences, Nanjing, China; ^4^State Key Laboratory for Biology of Plant Diseases and Insect Pests, Institute of Plant Protection, Chinese Academy of Agricultural Sciences, Beijing, China

**Keywords:** cotton, *Verticillium dahliae*, resistance, lysin-motif receptor kinases, VIGS

## Abstract

Lysin-motif (LysM) receptor kinases (LYKs) play essential roles in recognition of chitin and activation of defense responses against pathogenic fungi in the model plants *Arabidopsis* and rice. The function of LYKs in non-model plants, however, remains elusive. In the present work, we found that the transcription of two LYK-encoding genes from cotton, *Gh-LYK1* and *Gh-LYK2*, was induced after *Verticillium dahliae* infection. Virus-induced gene silencing (VIGS) of *Gh-LYK1* and *Gh-LYK2* in cotton plants compromises resistance to *V. dahliae*. As putative pattern recognition receptors (PRRs), both Gh-LYK1 and Gh-LYK2 are membrane-localized, and all three LysM domains of Gh-LYK1 and Gh-LYK2 are required for their chitin-binding ability. However, since Gh-LYK2, but not Gh-LYK1, is a pseudo-kinase and, on the other hand, the ectodomain (ED) of Gh-LYK2 can induce reactive oxygen species (ROS) burst in planta, Gh-LYK2 and Gh-LYK1 may contribute differently to cotton defense. Taken together, our results establish that both Gh-LYK1 and Gh-LYK12 are required for defense against *V. dahliae* in cotton, possibly through different mechanisms.

## Introduction

Plants trigger immune responses upon recognition of conserved microbe-derived components, known as microbe-associated molecular patterns (MAMPs) or pathogen-associated molecular patterns (PAMPs), via pattern recognition receptors (PRRs) (Nürnberger and Brunner, 2002; Bittel and Robatzek, 2007; Boller and Felix, 2009; Macho and Zipfel, 2014). MAMPs are invariant structures originating from microbial cell components that are not host constituents. Several MAMPs, including bacterial lipopolysaccharide (LPS), peptidoglycan (PGN), and fungal chitin, have been shown to possess immunogenic activities in animals and plants (Aslam et al., 2009; Newman et al., 2013), suggesting evolutionary similarity of pattern recognition systems between these two kingdoms.

The lysine-motif (LysM) was first identified in secretory bacterial hydrolases and is known to be involved in bacterial cell wall biogenesis, modification, and degradation (Beliveau et al., 1991). Encompassing 42–48 amino acids, the LysM is a ubiquitous domain found in all living organisms except for *Archaea* (Bateman and Bycroft, 2000; Zhang et al., 2007, 2009). LysM-containing receptors have been shown to be involved in the recognition of glycans containing *N*-acetylglucosamine (Buist et al., 2008). In model plants such as *Arabidopsis thaliana* (*Arabidopsis*) and *Oryza sativa* (rice), LysM receptor kinases (LYKs) are, together with leucine-rich repeat receptor-like kinase, the best studied PRRs, and are crucial for innate immunity against fungi and bacteria (Miya et al., 2007; Wan et al., 2008, 2012; Fradin et al., 2009; Shimizu et al., 2010; Willmann et al., 2011; Shinya et al., 2012; Cao et al., 2014; Hayafune et al., 2014; Kouzai et al., 2014; Paparella et al., 2014). The ectodomain (ED) of plant LYKs usually contains a signal peptide (SP) and LysMs, while their intracellular domain (ID) is known to contain an active or inactive kinase domain (Gust et al., 2012). Previous studies have shown that LYKs are essential for plant recognition of chitin or Nod factors, leading to the activation of plant innate immunity or beneficial symbioses (Liang et al., 2013, 2014). In rice, the chitin oligosaccharide elicitor-binding protein CEBiP was firstly shown to be required for the activation of the chitin PRR signaling pathway (Kaku et al., 2006; Kaku and Shibuya, 2016). Subsequently, OsCERK1 was proven to interact with CEBiP to regulate chitin-triggered defense responses (Shinya et al., 2010). In *Arabidopsis*, LYKs, including CERK1, LYK4, and LYK5, act as chitin PRRs to regulate the chitin signaling pathway (Miya et al., 2007; Wan et al., 2012; Cao et al., 2014), while LYK3 is involved in the negative regulation of immunity (Liang et al., 2013; Paparella et al., 2014). Two recent studies demonstrated that dimerization of CERK1 or CEBiP is required to activate immune signaling (Liu et al., 2012; Hayafune et al., 2014). Notably, the ED of CERK1, which is shed from the receptor, is involved in cell death regulation (Petutschnig et al., 2014). Although the functions of LYKs in model plants are gradually being revealed, the roles of LYKs in cotton defense against pathogens remain unknown.

Cotton (*Gossypium* spp.) is an important crop used in fiber, oil, and biofuel products. Four important cotton genera are cultivated around the world, including two allotetraploids (*Gossypium hirsutum* and *Gossypium barbadense*) and two diploids (*Gossypium herbaceum* and *Gossypium arboreum*). The allotetraploid cottons, especially *G. hirsutum*, are globally cultivated due to their good fiber quality and high yield. *Verticillium* wilt, primarily caused by the soil-born fungus *Verticillium dahliae* (Fradin and Thomma, 2006), is one of the most devastating plant diseases worldwide and is a major threat to cotton yield and fiber quality. *V. dahliae* from infected cotton can be categorized into two subgroups: defoliating and non-defoliating isolates (Daayf et al., 1995). The defoliating isolates cause severe leaf wilting, shedding, and cell death in the infected plants, whereas the non-defoliating isolates induce leaf-wilting symptoms with only minor leaf defoliation during the disease progression (Daayf et al., 1995; Zhang et al., 2012, 2013). *V. dahliae* isolates BP2 and V991 are non-defoliating and defoliating isolates, respectively, found in China (Zhang et al., 2012).

Our development of a cotton leaf crumple virus (CLCrV)-based virus-induced gene silencing (VIGS) vector has been proven to be an efficient gene-silencing tool that can be used for reverse genetics and functional analyses of candidate genes in cotton (Zhang et al., 2012; Gu et al., 2014; Liu et al., 2014, 2015; Chen et al., 2015; Fu et al., 2015; Zhang H. et al., 2016; Zhang W. et al., 2016). In this study, we employed CLCrV-based VIGS methods to study the function of two members of the cotton LYK family, *Gh-LYK1* and *Gh-LYK2*, in defense. Our results show that both genes are involved in chitin signaling perception and play important roles in resistance to *V. dahliae*. Furthermore, our findings indicate that Gh-LYK2 is involved in the regulation of cell death.

## Materials and methods

### Plant materials, *V. dahliae* isolates, and culture conditions

Cotton (*G. hirsutum*) cultivars 3503 and ZN905 were cultivated at temperatures ranging from 20 to 25°C, under a 16 h:8 h, light: dark photoperiod at 80% relative humidity. The defoliating isolate V991 and the non-defoliating isolate BP2 of *V. dahliae* were maintained on potato dextrose agar (PDA) at 25°C for 7 days, followed by inoculation into Czapek's medium in 1 l of distilled water (Zhang et al., 2012). The conidial suspensions of *V. dahliae* were adjusted to 1 × 10^6^ conidia ml^−1^ with distilled water and used for the inoculation of plants.

### Gene cloning

Full-length ORFs of *Gh-LYK1, Gh-LYK2*, and *Gh-LYK5* were amplified from the *G. hirsutum* cultivar 3503 cDNA with three primer pairs: LYK1 full F and LYK1 full R; LYK2 full F and LYK2 full R; and LYK5 full F and LYK5 full R, respectively (Table S1). The PCR products were purified via gel electrophoresis and cloned into the pZeroback/blunt plasmid (Tiangen) to produce pLYK1, pLYK2, and pLYK5.

### VIGS vector construction, agroinfiltration, and *V. dahliae* inoculation

To generate the VIGS vector for silencing of *Gh-LYK1*, a 325-bp fragment of Gh-LYK1 was amplified from pLYK1 via PCR with the primer pair LYK1 F *Spe*I and LYK1 R *Pac*I (Table S1). The resulting PCR product was inserted into pCLCrVA to produce pCLCrV-LYK1. Another 333-bp fragment was amplified from pLYK2 with the primer pair LYK2 F *Spe*I and LYK2 R *Avr*II (Table S1), and the fragment was inserted into the *Spe*I-*Avr*II sites of pCLCrVA to produce pCLCrV-LYK2.

To generate a double-silencing vector for *Gh-LYK1* and *Gh-LYK2*, two primers (LYK2 silencing F *Pac*I and LYK2 silencing R *Asc*I) (Table S1) were used for PCR amplification from the pCLCrV-LYK2 template. The PCR products were purified, digested with *Pac*I and *Asc*I and inserted into pCLCrV-LYK1 to produce pCLCrV-LYK1+2. All of the plasmids were sequenced; the primers used are listed in Table S1.

Transformed *Agrobacterium* cultures were grown to an approximate OD600 of 1.0 and incubated in induction buffer [10 mM MgCl_2_, 100 mM MES (pH 5.7), 100 μM acetosyringone] for 3 h at room temperature. Combinations of the induced cultures were infiltrated into the abaxial side of leaves of four-leaf-stage *Nicotiana benthamiana* plants or the abaxial side of cotyledons of 2-week-old cotton seedlings.

After agro-infiltration, the cotton plants were maintained in an incubator at 25°C with 80% relative humidity. At 15 dpi, all VIGS-treated plantlets were evaluated for CLCrV replication via PCR with the specific primers CLCrV-F and CLCrV-R. More than 15 positive plants per target were then inoculated with *V. dahliae* through soil drenching with 30 ml of a conidial suspension (1 × 10^6^ conidia ml^−1^) in each pot (250 ml). Foliar damage was evaluated by rating the symptoms on the cotyledons and leaves of inoculated plants according to the following disease grades: 0 = healthy plant; 1 = yellowing or necrosis of 1–2 cotyledons; 2 = yellowing or necrosis of 1 true leaf; 3 = more than two wilted or necrotic leaves; and 4 = all leaves wilted or dead plant. The disease index was calculated according to the following formula: disease index = [∑disease grades × number of infected)/(total plants × 4)] × 100 (Zhang et al., 2012). The VIGS experiments and the *V. dahliae* infection assays were repeated three times with more than 15 plants analyzed per replicate.

### PAMP treatment

The roots of 2-week-old cotton seedlings were immersed in PAMP solutions containing 0.1% Tween-20 and 10 μg ml^−1^ PGN (Sigma), 10 μg ml^−1^ chitin (Sigma), 10 μg ml^−1^ LPS (Sigma), or 1 μM flg22 (Phytotech, USA), and were then subjected to vacuum while shaking at 90 rpm for 30 min.

### Quantitative RT-PCR

The primers for quantitative RT-PCR were designed using the Primer5 software. The specificity of the primers was evaluated by subjecting the primer sequences to BLAST searches against the NCBI database. PCR amplification was performed in a qTOWER 2.0/2.2 real-time thermal cycler (Analytikjena, Germany) with a 25 μl final volume containing 2.5 μl of cDNA, 0.5 μl of each primer (10 μM), 9 μl of sterile water, and 12.5 μl (2 ×) of SYBR Premix ExTaqTM II Kit (TaKaRa). The conditions for amplification were as follows: 5 min denaturation at 95°C followed by 40 cycles of 95°C for 10 s, 60°C for 20 s, and 72°C for 10 s. The expression levels of selected genes were normalized to that of *UBQ14*, and relative gene expression was calculated using the 2^−ΔΔCT^ method. Data are presented as the mean ± standard error (SE) of three independent experiments in which three independent plants were included per experiment. The primer sequences are listed in Table S1.

### *In planta V. dahliae* biomass quantification

Quantification of *V. dahliae* biomass was performed as described previously (Fradin et al., 2011). Essentially, four *V. dahliae* inoculated plants per genotype were harvested and pooled. The samples were ground into fine powder in liquid nitrogen, and DNA was extracted from 100 mg of powder. *V. dahliae* biomass was determined by real-time PCR. To assess *V. dahliae* biomass, the fungus specific ITS1-F primer and *V. dahliae*-specific reverse primer ST-Ve1-R (Fradin et al., 2011) were used. The primer pair AtRuBisCo-F3 and AtRuBisCo-R3, targeting the large subunit of the Rubisco gene (Fradin et al., 2011) in *Arabidopsis*, and the primer pair UBQ14F and UBQ14R, targeting the *UBQ14* gene in cotton (Table S1) were used for sample calibration, respectively.

### Subcellular localization analysis

To evaluate the subcellular localization of the Gh-LYKs, the full-length cDNAs of *Gh-LYK1/2* were generated via PCR. The gene encoding At-PIP2A (AT3G53420), which is used as a plasma membrane (PM) marker (Cutler et al., 2000), was amplified from an *Arabidopsis* cDNA and cloned into the pCHF3-dsRED vector. The plasmids were individually introduced into *Agrobacterium tumefaciens* strain EHA105. To enhance ectopic expression, agrobacteria harboring TBSV P19 were co-infiltrated into tobacco leaves. The co-localization of GFP-labeled Gh-LYK derivative fusion proteins and dsRED-labeled At-PIP2A fusion proteins was analyzed with a ZEISS confocal microscope (LSM 780, Germany) at 72 h post-infiltration.

### Protein expression and purification

To enhance the solubility of proteins expressed in *Escherichia coli* (*E. coli*), a pGTf2 plasmid (TAKARA) encoding chaperons was introduced into *E. coli* strain DE3 to produce strain DE3-Tf2. All of the prokaryotic expression assays described below were performed in this strain.

The EDs of Gh-LYK1 and Gh-LYK12 (minus signal peptide) were cloned into pET30a to form 6 × His tag fusion proteins. The fusion proteins were purified with Ni-NTA resin (Novagen) and used in the chitin-binding assay. To test the kinase activities of Gh-LYK1 and Gh-LYK2, the internal regions of Gh-LYK1 (residues 253–620) and Gh-LYK2 (residues 286–672) from cultivar 3503 were cloned into pMBP28 to form N-terminal maltose-binding protein (MBP) tag fusion proteins, designated Gh-LYK1-ID and Gh-LYK2-ID, respectively. The fusion proteins were then purified over amylose resin (New England Biolabs) and used in the kinase assays.

### Chitin-binding assays

The *in vitro* chitin binding assay was performed as described previously (Petutschnig et al., 2010) with minor modifications. Each purified protein was incubated with the chitin beads (New England Biolabs) at 4°C on a rocking platform for 3h. The mixtures were then centrifuged for 5 min at 13,000 g. The pellet fractions were rinsed five times with CBD binding buffer (500 mM NaCl, 20 mM Tris-HCl, 1 mM EDTA 0.1% Tween-20, pH8.0 25°C). Both the pellet and supernatant fractions collected after the last rinse were boiled in SDS loading buffer for 10 min, and centrifuged for 10 min at 13,000 g. Twenty milliliters of supernatant fractions of the solutions were used to perform 12% SDS-PAGE and immunoblot analysis with anti-His antibody (Tiangen, Beijing, China).

Additionally, the EDs of Gh-LYK1, Gh-LYK2 and derivatives were transiently expressed in *N. benthamiana* leaves. At 72 h post-agroinfiltration, the infiltrated leaves were harvested. Homogenization buffer [50 mM HEPES-KOH (pH 7.4), 150 mM NaCl, 5 mM NaF, 1 mM Na_3_VO_4_, 0.5% Triton X-100, 1 mM PMSF, 1 × PI cocktail (Roche)] was added at 2 ml per g of plant material. The sample was then centrifuged with 5,000 × *g* at 4°C for 15 min, and the supernatant mixture was incubated with chitin beads at 4°C for 3 h. The incubated beads were rinsed five times with the homogenization buffer. The finally rinsed beads were subsequently boiled in SDS loading buffer for 10 min and centrifuged for 10 min at 13,000 g. The supernatant fractions of the solutions were used to perform SDS-PAGE and immunoblot analyses with an anti-Flag antibody (Sigma).

### Kinase assays

For the kinase assays, the purified Gh-LYK1-ID or Gh-LYK2-ID protein was individually incubated with γ-^32^P ATP at 30°C for 30 min, as previously described (Liu et al., 2011). The phosphorylated substrate was visualized with a Typhoon 9200 imager (GE Healthcare) after separation through SDS-PAGE.

### DUAL membrane yeast two-hybrid system

To detect interactions between Gh-LYK1 and Gh-LYK2 in yeast, the full-length ORFs of *Gh-LYK1* and *Gh-LYK2* were amplified from pLYK1 and pLYK2 and cloned into pBT3-C and pPR3-C, respectively. DUAL membrane yeast two-hybrid assays were then performed according to the manufacturer's protocol (Dualsystems Biotech, Switzerland).

### Homology model building

Homology modeling was performed to generate the 3D structure of Gh-LYK1. The crystal structure of the ED of a receptor-like kinase from *Arabidopsis*, which shares 51% sequence identity with Gh-LYK1, in complex with a chitin pentamer (PDB ID: 4EBZ) was used as template. Gh-LYK1 homology models were generated using Modeller 9.16 (Martí-Renom et al., 2000; Webb and Sali, 2016).

### Reactive oxygen species (ROS) and cell-death detection *in planta*

The accumulation of H_2_O_2_ was detected using 3,3′-Diaminobenzidine as previously described, with minor modifications (Qian et al., 2015). Briefly, leaves were excised at the base of the petiole and supplied with a 1 mg/ml solution (pH 3–3.8) of DAB (Sigma-Aldrich) for 8 h at 25°C. Leaves were then immersed in boiling 96% ethanol for 5–10 min, cooled and preserved at room temperature in 70% ethanol, and photographed.

The trypan blue staining assay was performed as described previously, with minor modifications (Heese et al., 2007). Briefly, leaves were submerged in trypan blue staining solution (6 vol of ethanol, 1 vol of distilled water, 1 vol of lactic acid, 1 vol of glycerol, 1 vol of phenol, and 0.067% w/v trypan blue) for 45 min without boiling. After staining, leaves immersed in ethanol were shaken overnight at room temperature until the tissue had become completely colorless.

### Bimolecular fluorescence complementation (BiFC) assay

To detect the dimerization of Gh-LYK1/2 in tobacco leaves, the full length ORFs of *Gh-LYK1* and *Gh-LYK2* were cloned into p2YN and p2YC, respectively. The resulting plasmids were individually introduced into *A. tumefaciens* strain EHA105 by electroporation using a Gene Pulser Apparatus (Bio-Rad, Hercules, CA) as described (Huang et al., 2009). The bimolecular fluorescence complementation (BiFC) assay was then performed as described previously (Yang et al., 2007). To enhance the ectopic expression, Agrobacterium harboring the gene silencing suppressor P19 was co-infiltrated into tobacco leaves. Emission of YFP fluorescence was detected and the cells were imaged under a confocal microscope (LSM 780, Germany) at 48 h post infiltration.

## Results

### *Gh-LYK1* and *Gh-LYK2* are up-regulated during *V. dahliae* infection and chitin treatment

**Figure 1:**
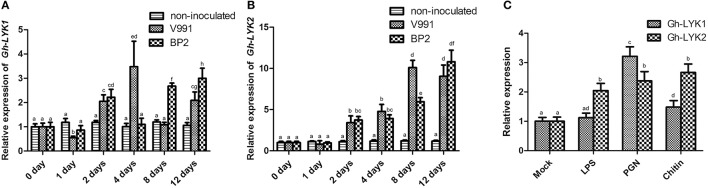
Expression of Gh-LYK1 and Gh-LYK2 is induced by challenge with *V. dahliae*. **(A,B)** Relative expression of Gh-LYK1 **(A)** and Gh-LYK2 **(B)** in roots determined through quantitative reverse transcription PCR (qRT-PCR) at 0, 1, 2, 4, 8, 12 days post *V. dahliae* inoculation. Values with the same lower case letter above the error bar were not significantly different according to Duncan's multiple range tests (*P* < 0.05). **(C)** Relative expression of Gh-LYK1 and Gh-LYK2 in roots determined through qRT-PCR in cotton treated with various PAMPs. Values with the same lower case letter above the error bar were not significantly different according to Duncan's multiple range tests (*P* < 0.05).

There are five LYK gene family members encoded in the *Arabidopsis* genome (At-LYK1–At-LYK5) (Zhang et al., 2007, 2009). To identify the homologs of *Arabidopsis* LYKs in cotton (*G. hirsutum*), we used the full-length open reading frames (ORFs) of *At-LYK1* to *At-LYK5* as queries in BLAST searches against the *Gossypium* unigene database (https://phytozome.jgi.doe.gov) and retrieved the candidates with highest BLAST scores. We identified two *LYK1* homologs (D_cotton_018813, D_cotton_000932), one *LYK2* homolog (D_cotton_035752), one *LYK3* homolog (D_cotton_014206), two *LYK4* homologs (D_cotton_017682, D_cotton_017683), and one *LYK5* homolog (D_cotton_019720) encoded in the *Gossypium* genome. We monitored the expression of these seven putative *Gh-LYK* genes in the roots of the *V. dahliae* resistant cultivar 3503 during infections with defoliating and non-defoliating *V. dahliae* isolates. The expression of D_cotton_018813, D_cotton_014206, D_cotton_017682, and D_cotton_017683 in roots was too low to be detected (data not shown), while the expression of D_cotton_000932, D_cotton_035752, and D_cotton_019720 was up-regulated during *V. dahliae* infection (Figures 1A,B and Figure S1). Thus, these three putative genes (D_cotton_000932, D_cotton_035752, and D_cotton_019720) were designated as *Gh-LYK1, Gh-LYK2*, and *Gh-LYK5*, respectively, for further study. As the *Gh-LYK5*-silenced cotton plants exhibited a similar *V. dahliae* biomass compared with control plants after challenge with *V. dahliae* (Figure S2), we focused on the functional characterization of *Gh-LYK1* and *Gh-LYK2*. The expression of *Gh-LYK1* and *Gh-LYK2* increased significantly at 2 days post inoculation with *V. dahliae*. At 12 days post inoculation, the relative mRNA levels of *Gh-LYK1* and *Gh-LYK2* increased by at least 2 and 9-fold, respectively, while there were no significant changes in the levels of *Gh-LYK1* or *Gh-LYK2* transcription in the non-inoculated control (Figures 1A,B). To define whether the expression of *Gh-LYK1* and *Gh-LYK2* was responsive to MAMPs, we analyzed the transcription of *Gh-LYK1* and *Gh-LYK2* in roots treated with Flg22, LPS, PGN, and chitin. As expected, the expression of *Gh-LYK1* or *Gh-LYK2* was up-regulated by both PGN and chitin treatments (Figure 1C and Figure S3A). Interestingly, LPS also induced the expression of *Gh-LYK2* (Figure 1C). On the contrary, we did not see any significant changes in the transcription of *Gh-LYKs, WRKY53*, and *MPK3* in cotton roots after a 30-min treatment with Flg22 (Figure S3A), or even a 24-h treatment (Figure S3B). The expression changes of *Gh-LYK1* and *Gh-LYK2* suggested that both of these LYKs might be involved in chitin-triggered immunity during the defense against *V. dahliae* in cotton.

Next, we cloned the full-length cDNAs of *Gh-LYK1* and *GhLYK2* from *G. hirsutum* cultivar 3503. The ORF of *Gh-LYK1* and *Gh-LYK2* are 1,860 and 2,016 bp, respectively (GenBank accession numbers KU598832 and KU598833). Both of these proteins share a similar domain structure with an SP, extracellular LysM domains, a transmembrane (TM) domain, and an intracellular Ser/Thr kinase domain. Phylogenetic analysis and amino acid (aa) sequence alignment revealed that Gh-LYK1 shares 61.4% identity with At-LYK1, while Gh-LYK2 shares 41% identity with At-LYK2 (Figure S4).

### Silencing of *Gh-LYK1* and *Gh-LYK2* compromises resistance to *V. dahliae* in cotton

**Figure 2:**
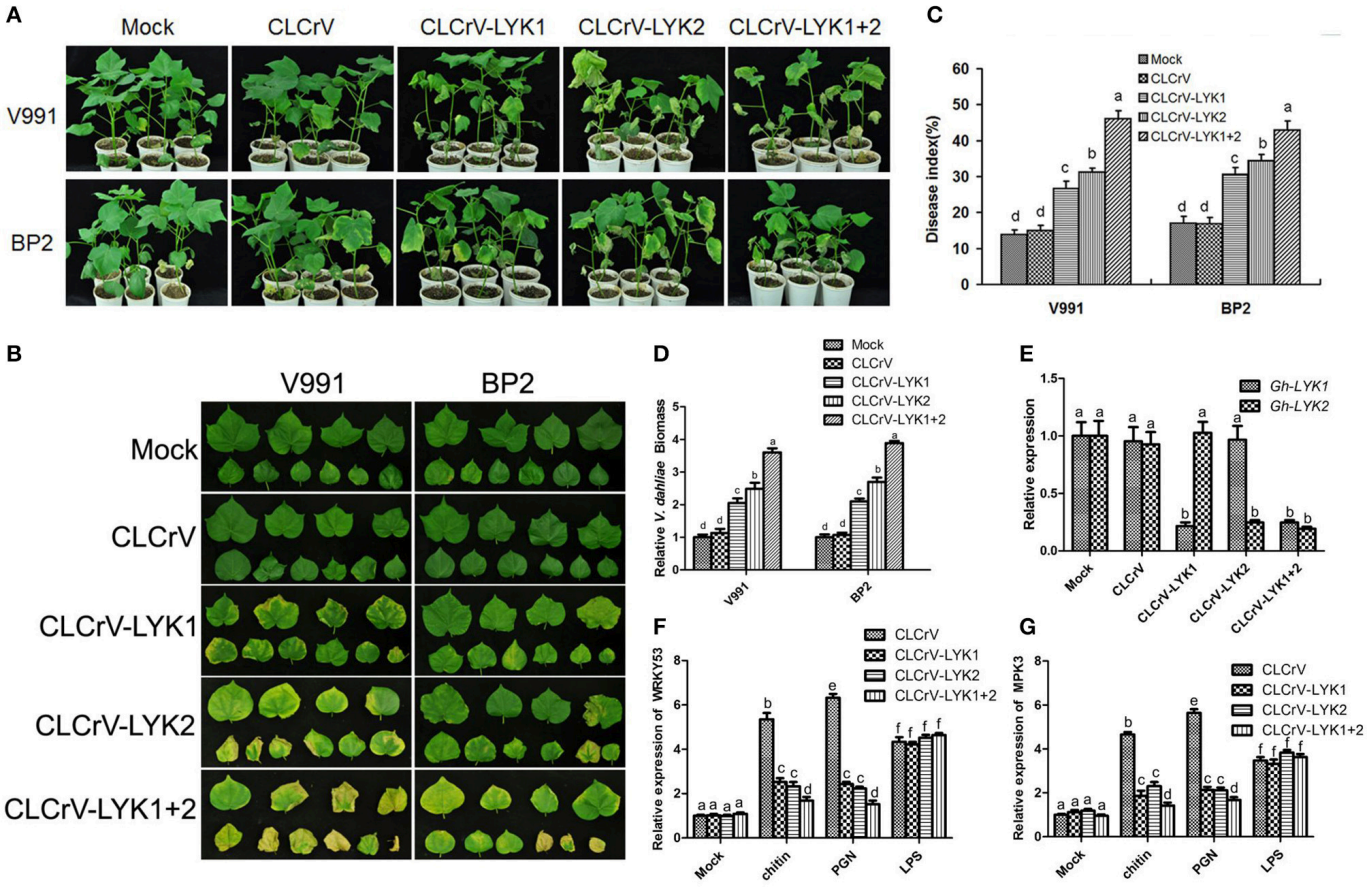
Silencing of Gh-LYK1 and Gh-LYK2 enhances the susceptibility of cultivar 3,503 to *V. dahliae*. **(A,B)** Typical symptoms of Gh-LYK1-silenced or Gh-LYK2-silenced whole plants **(A)** and leaves **(B)** in which Gh-LYK1 and Gh-LYK2 were simultaneously silenced, infected with two isolates of *V. dahliae* at 30 days post *V. dahliae* inoculation. Mock- and empty vector-infiltrated plants were used as controls. **(C)** Disease index of the silenced plants and non-silenced plants challenged with *V. dahliae* at 30 days post *V. dahliae* inoculation. The experiment was repeated three times. The error bars represent SE of the biological replicates. Values with the same letter were not significantly different according to Duncan's multiple range tests (*P* < 0.05). **(D)** The *V. dahliae* biomass in the silenced and non-silenced plants challenged with *V. dahliae* was estimated at 30 days post *V. dahliae* inoculation. The error bars represent SE of the biological replicates. Values with the same lower case letter above the error bar were not significantly different according to Duncan's multiple range tests (*P* < 0.05). **(E)** Relative expression of Gh-LYK1 and Gh-LYK2 in the roots of the silenced and non-silenced plants measured through qRT-PCR. Values with the same letter were not significantly different according to Duncan's multiple range tests (*P* < 0.05). **(F,G)** Relative expression of *WRKY53* **(F)** and *MPK3* **(G)** in the silenced and non-silenced plants measured through qRT-PCR. Roots from empty vector-infiltrated or VIGS-silenced seedlings were incubated with PAMPs or sterile water (mock) for 30 min and the induction of *WRKY53* and *MPK3* was determined by qPCR. Values with the same letter were not significantly different according to Duncan's multiple range tests (*P* < 0.05).

To determine the roles of *Gh-LYK1* and *Gh-LYK2* in defense, we used the CLCrV-based VIGS system to silence the transcription of *Gh-LYK1* or *Gh-LYK2* in cotton and tested the defense responses of the silenced plants upon *V. dahliae* infection. We generated the vectors pCLCrV-LYK1, pCLCrV-LYK2, and pCLCrV-LYK1+2, which can silence *Gh-LYK1, Gh-LYK2*, or both, in the VIGS system, respectively. Agrobacteria containing CLCrV, CLCrV-LYK1, CLCrV-LYK2, or CLCrV-LYK1+2 were infiltrated into the cotyledons of cotton cultivar 3503. At 15 days post-infiltration (dpi), silenced and non-silenced plants were challenged with *V. dahliae* isolates V991 or BP2. At 30 days post *V. dahliae* inoculation, we scored the disease index in the VIGS silenced plants using mock- and empty vector-infiltrated plants as controls. In general, the CLCrV-LYK1+2-silenced cotton plants were more susceptible to the infection than the rest (Figures 2A,B). Notably, defoliating strain V991 is more virulent than BP2 in most cotton cultivars, but not in cultivar 3503. After challenge with V991, the average disease index of the CLCrV-LYK1-, CLCrV-LYK2-, and CLCrV-LYK1+2-infiltrated plants were 27, 31, and 46, respectively, whereas the index of the mock- and CLCrV-infiltrated plants were 14 and 15, respectively. For the inoculation of non-defoliating isolate BP2, the average disease index of the CLCrV-LYK1-, CLCrv-LYK2-, and CLCrV-LYK1+2-infiltrated plants were 31, 34, and 42, respectively, whereas the index in both the mock- and CLCrV-infiltrated plants was 17 (Figure 2C). To evaluate the pathogen propagation in cotton plants, the biomass of *V. dahliae* was measured by qPCR, and the results were correlated with the disease index (Figure 2D). Silencing of the specific genes was confirmed via qRT-PCR analysis using RNA isolated from the roots (Figure 2E). Additionally, we monitored the expression of two defense-related genes, *WRKY53* and *MPK3* (Murray et al., 2007; Meng et al., 2013), in MAMP-treated Gh-LYK1/2/1+2-silenced or mock cotton plants. The inducible transcription of both *WRKY53* and *MPK3* by chitin or PGN was significantly decreased in the silenced cotton plants as compared to the controls (Figures 2F,G). These results indicate that *Gh-LYK1* and *Gh-LYK2* are important for basal resistance in cotton, and that they act additively.

### Subcellular localization of Gh-LYK1 and Gh-LYK2

**Figure 3:**
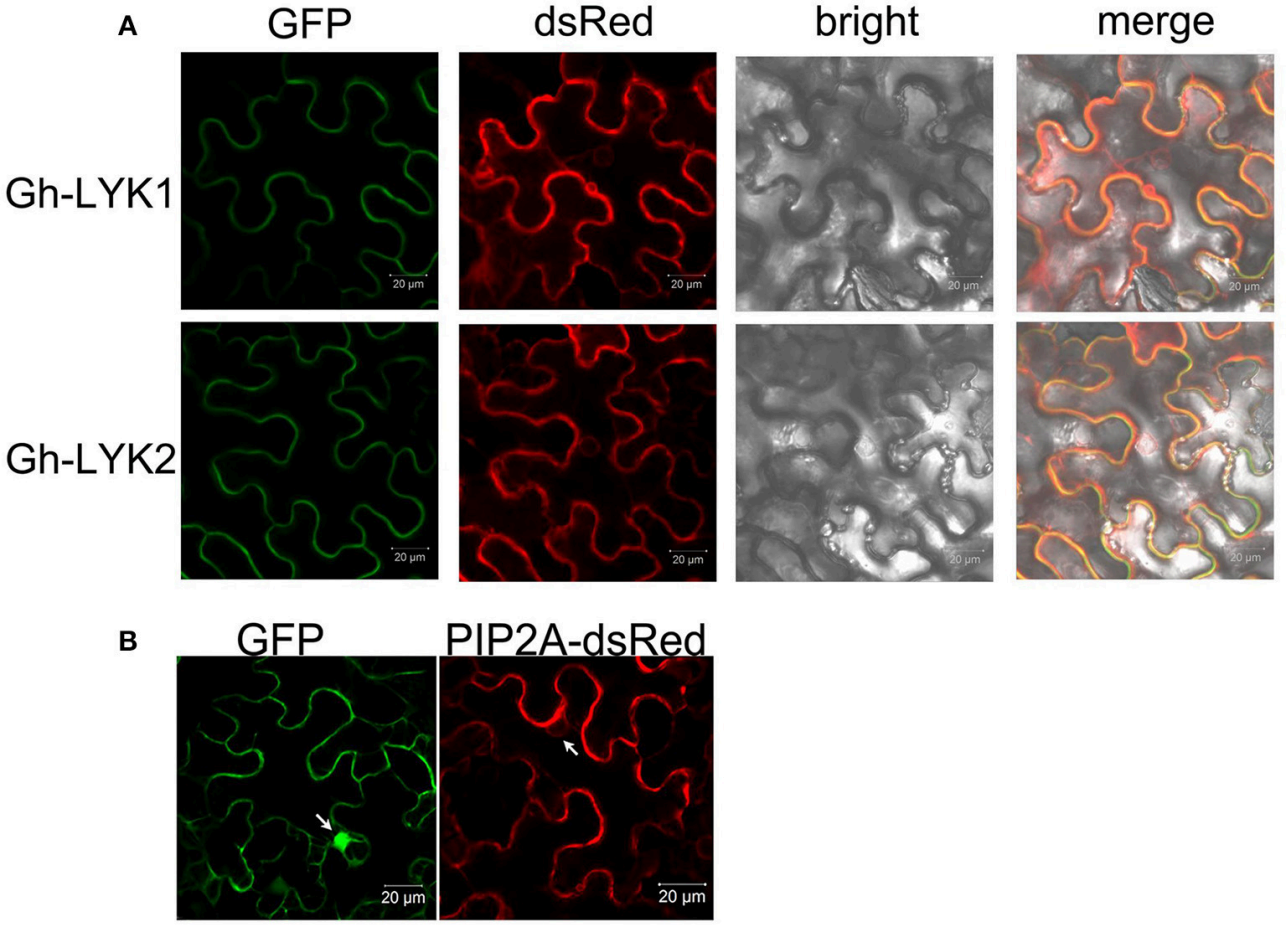
The subcellular localization of Gh-LYK1 and Gh-LYK2 **(A)** Confocal microscopy images of tobacco leaf cells infiltrated with *Agrobacterium* harboring the Gh-LYK1-GFP and Gh-LYK2-GFP fusion constructs. Fluorescent signals from AtPIP2A-dsRed fusion proteins were used as the membrane marker. **(B)** Fluorescent images of GFP alone and AtPIP2A-dsRed fusion proteins under a confocal microscope. White arrows indicate the nuclei.

To investigate the subcellular localization of Gh-LYK1/2, we generated constructs to express the full-length sequences of Gh-LYK1 or Gh-LYK2 fused to the green fluorescent protein (*GFP*) at their C-terminus and driven by the CaMV 35S promoter, designated as Gh-LYK1-GFP and Gh-LYK2-GFP, and then transiently expressed in *N. benthamiana* leaves. Under the confocal microscope, the green florescence signal of Gh-LYK1-GFP or Gh-LYK2-GFP was localized to the PM (Figure 3A). To confirm these localization, an AtPIP2A-dsRed fusion construct, which has been used as a PM marker (Pumplin and Harrison, 2009; Pumplin et al., 2012; Sun et al., 2013), was co-expressed with Gh-LYK-GFP. Gh-LYK1/2-GFP co-localized with AtPIPA2-dsRed (Figure 3A), while GFP alone did not (Figure 3B). To further confirm their PM localization, we observed Gh-LYK1-GFP and Gh-LYK2-GFP in plasmolysed *N. benthamiana* leaves. After plasmolysis with 30% glycerol, Gh-LYK1-GFP and Gh-LYK2-GFP were visualized at the PM and in Hechtian strands (Figure S5).

### All three LysMs of Gh-LYK1 and Gh-LYK2 are required for their chitin-binding ability

**Figure 4:**
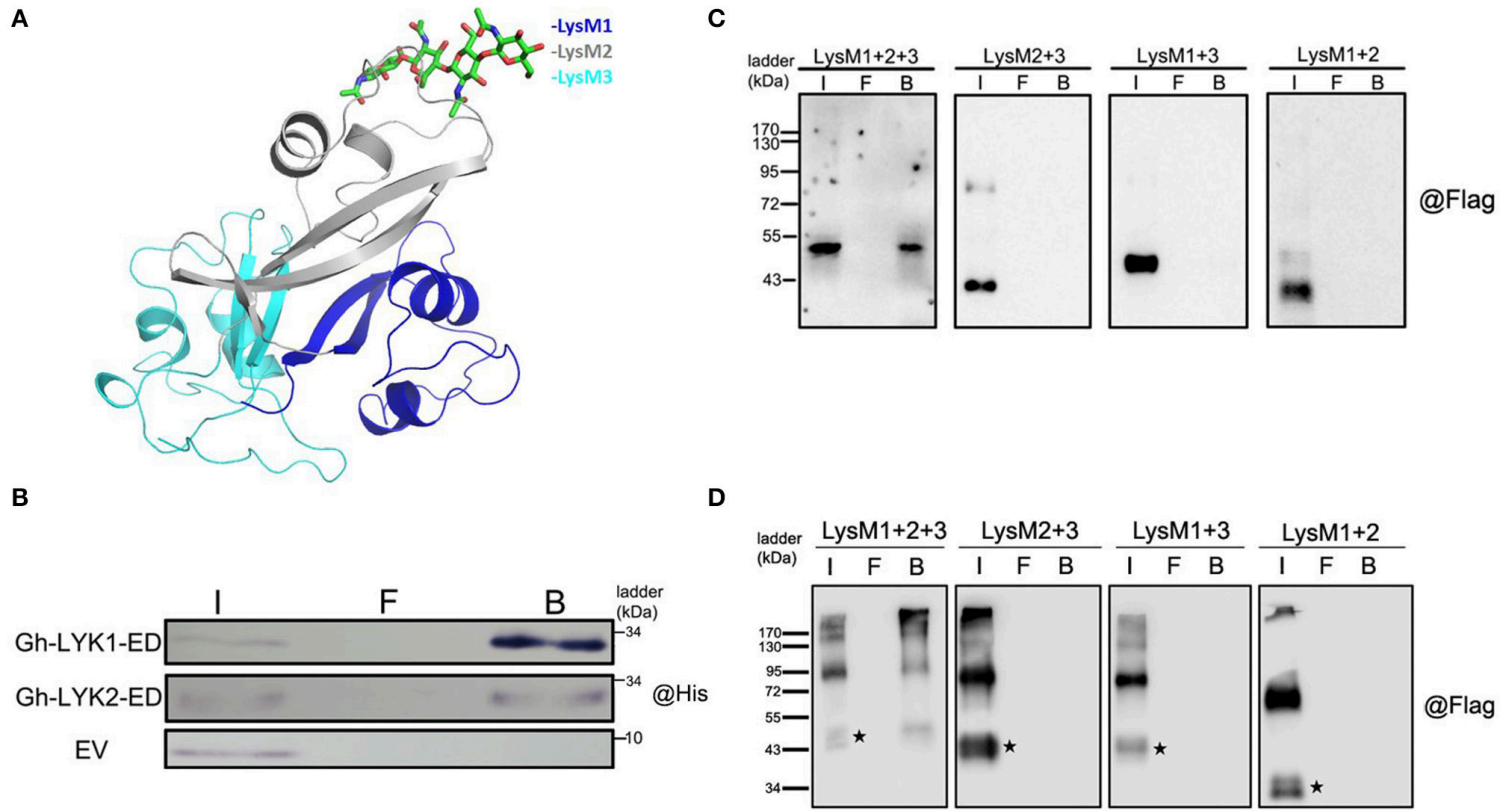
Modeled structure of Gh-LYK1 with chitin and chitin-binding assay. **(A)** Computer modeling of tight packing of the three LysM domains in Gh-LYK1 with chitin. Chitin is shown in green sticks. **(B)** Chitin beads binding assay of purified Gh-LYK1-ED and Gh-LYK2-ED. I: input, F: flow through, B: beads. **(C)** Chitin beads binding assay of Gh-LYK1-ED and single LysM domain deletion mutants expressed in *N. benthamiana* leaves. I: input, F: flow through, B: beads. **(D)** Chitin beads binding assay of Gh-LYK2-ED and single LysM domain deletion mutants expressed in *N. benthamiana* leaves. I: input, F: flow through, B: beads. The black pentagram indicates the monomer.

Because the ED of Gh-LYK1 shares 51% sequence similarity with that of the chitin receptor kinase of *Arabidopsis* (AtCERK1-ED), we performed homology modeling of Gh-LYK1-ED based on a crystal structure of AtCERK1-ED in complex with chitin residues (PDB ID: 3KTI). As shown in Figure 4A, all of the three LysM domains of Gh-LYK1 pack tightly against each other, resulting in a globular structure that is similar to that of AtCERK1-ED. Previous publications have shown that all three LysMs are required for the chitin-binding ability of LYKs (Petutschnig et al., 2010; Cao et al., 2014). Hence, deletion of any LysM domain may disturb the structural integrity, resulting in loss of chitin-binding ability. Next, we determined the chitin-binding activities of Gh-LYK1 and Gh-LYK2 using *in vitro* chitin pull-down assays. The results showed that both purified recombinant EDs can directly bind to chitin beads (Figure 4B). To further confirm this result, we transiently expressed the full-length and LysM single-deletion Gh-LYKs-ED mutants fused to a C-terminal Flag-HA dual tag in *N. benthamiana* leaves and checked chitin-binding activity as previously described (Petutschnig et al., 2010). In accordance with the results of the *in vitro* assays, the full length Gh-LYK1-ED and Gh-LYK2-ED could bind chitin beads, while deletion of any of the LysM domains abolished their chitin-binding ability (Figures 4C,D).

### Gh-LYK2 forms chitin-independent dimers *in vivo*

**Figure 5:**
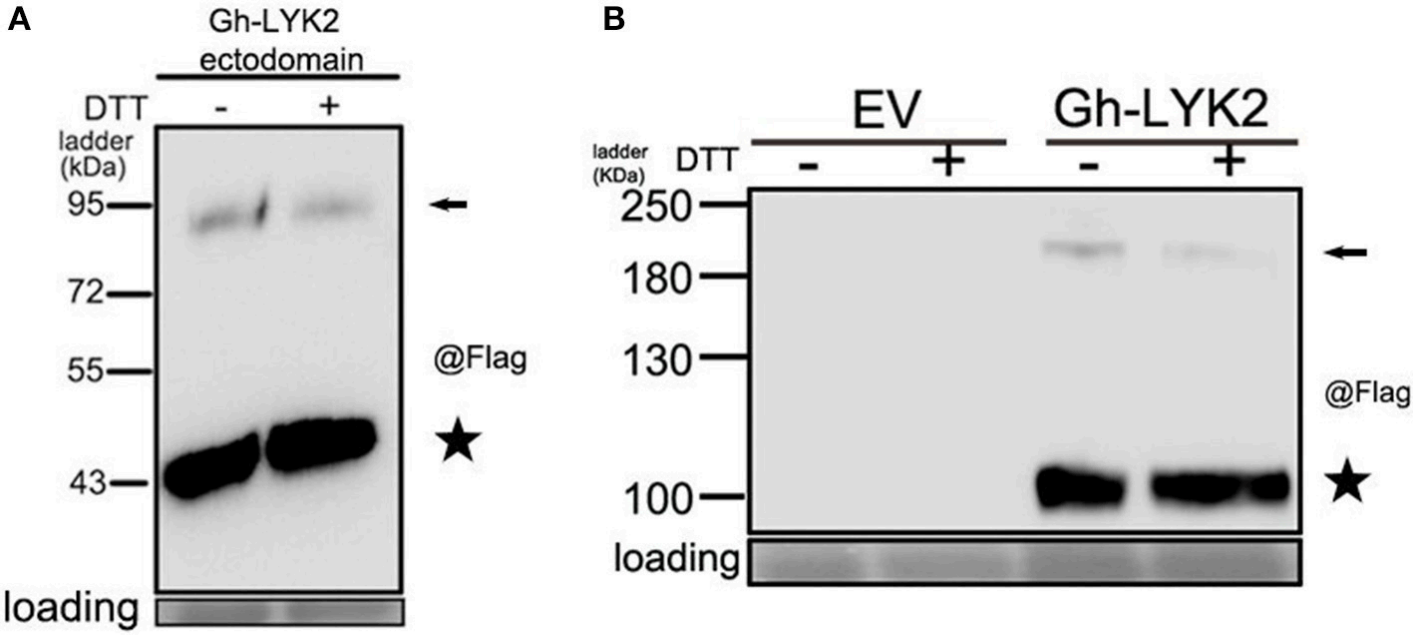
Gh-LYK2 forms dimer in *N. benthamiana* leaves. **(A)** The dimers of Gh-LYK2-ED were not disrupted by DTT. **(B)** The dimers of Gh-LYK2 were not disrupted by DTT. Black arrows indicate full-length dimer; black pentacles indicate the monomer.

Previous studies have shown that homodimerization of AtCERK1 and Os-CEBiP is important for chitin perception (Liu et al., 2012; Hayafune et al., 2014). Unexpectedly, we found that a portion of Gh-LYK2-ED formed dimers or oligomers in *N. benthamiana* without induction by chitin (Figure 4D). To determine whether disulfide bonds contribute to the formation of the dimer, total protein extracts were treated with extra 100 mM Dithiothreitol (DTT) and then analyzed via immunoblotting. The results show that the dimers were not disrupted by DTT (Figure 5A), which suggests that this dimer does not depend on disulfide bond linkages.

We next tested whether the full-length Gh-LYK2 could also form dimers in the absence of chitin. The full-length sequence of Gh-LYK2 was transiently expressed in *N. benthamiana* fused to a C-terminal Flag-HA dual tag. Similar results to those with Gh-LYK2-ED were obtained (Figure 5B). To test the dimerization of Gh-LYK2 *in vivo*, we performed a BiFC assay. The yellow fluorescent protein (YFP) signal, absent in controls, indicated formation of a Gh-LYK2 homo-dimer (Figure S6A). Taken together, these results indicate that the homodimerization of Gh-LYK2 is chitin-independent and does not involve disulfide linkages.

### Gh-LYK2, but not Gh-LYK1, contains a pseudo-kinase domain

**Figure 6:**
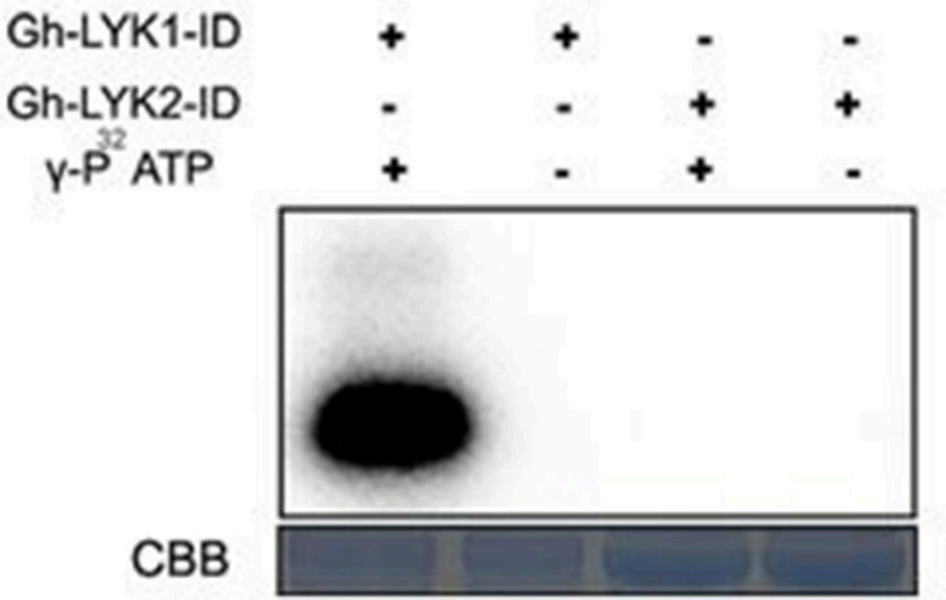
*In vitro* kinase activities of Gh-LYK1-ID and Gh-LYK2-ID. The auto-phosphorylation was measured by incorporation ofγ-^[32P]^-ATP. Up panel shows autoradiograph, and bottom panel shows gel stained with CBB. CBB, coomassie brilliant blue.

Previous reports have shown that Lotus LjNFR5, Medicago NFP, and *Arabidopsis* LYK4/5 all contain a dead kinase domain (Madsen et al., 2003; Radutoiu et al., 2003; Arrighi et al., 2006; Wan et al., 2012). We compared the sequence of the kinase domains of the Gh-LYKs to that of typical kinases and found that several key residues were altered or absent, especially in subdomains I, III, and VII (Figure S7) in Gh-LYK2. These differences suggest that Gh-LYK2 is an atypical kinase. On the contrary, the Gh-LYK1 kinase domain is very similar to that of At-LYK1 in its subdomain alignment as well as in the key catalytic amino acid residues (Figure S7). To test whether Gh-LYK1-ID and Gh-LYK2-ID are active kinases, we expressed and purified Gh-LYK1-ID and Gh-LYK2-ID from *E. coli* and performed *in vitro* kinase assays. The results showed that, unlike Gh-LYK1-ID, Gh-LYK2-ID was not capable of catalyzing auto-phosphorylation (Figure 6). We next tested whether Gh-LYK2 can interact with Gh-LYK1: no interaction could be detected either in BiFC or dual membrane yeast two-hybrid assays (Figures S6A,B, S8).

### Overexpression of Gh-LYK2-ED induces a burst of reactive oxygen species in *N. benthamiana*

**Figure 7:**
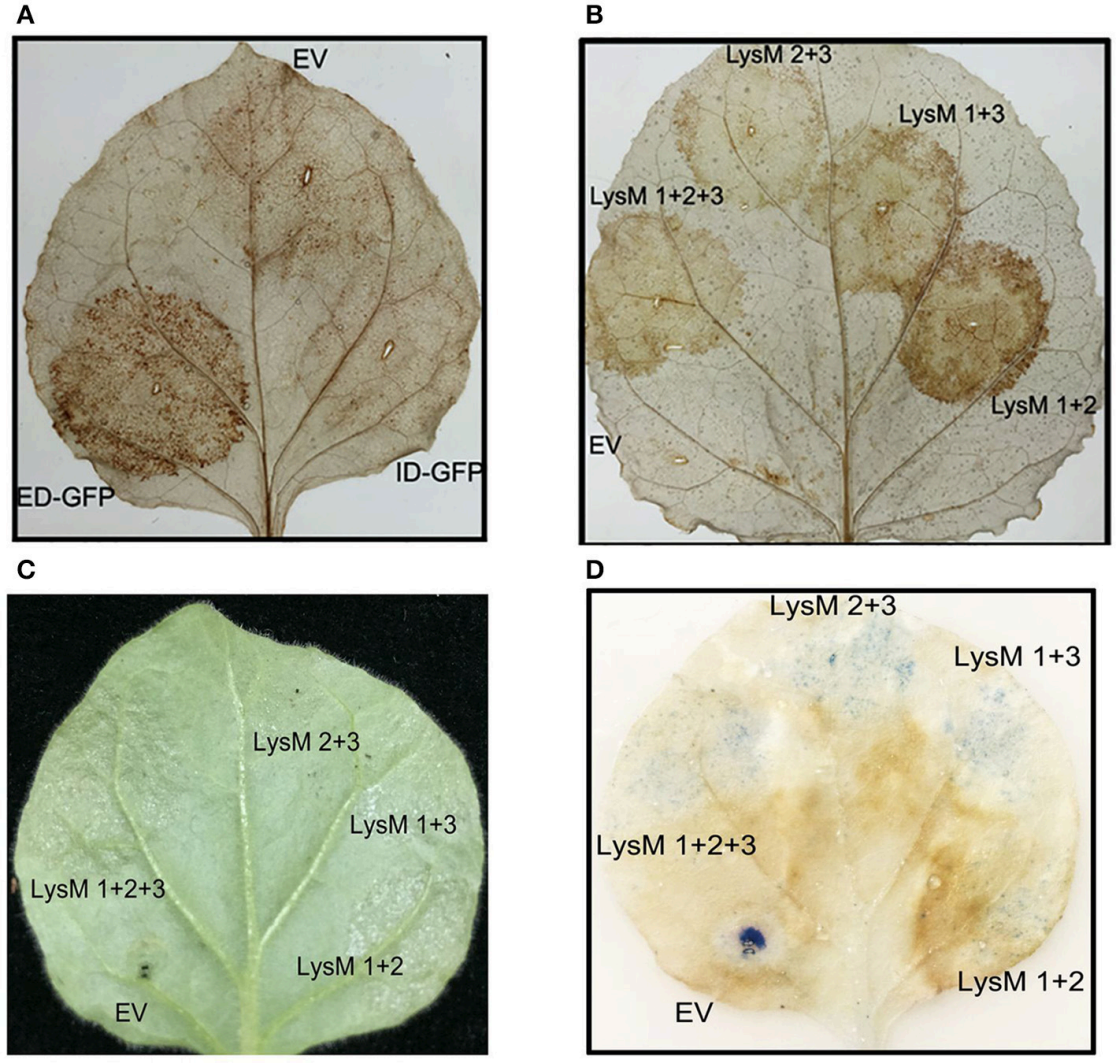
Transient expression of Gh-LYK2-ED can induce the accumulation of ROS and cell death of in *N. benthamiana*. **(A)** DAB staining of *N. benthamiana* leaf expression with Gh-LYK2-ED-GFP, Gh-LYK2-ID-GFP, and EV. EV, empty vector. **(B)** DAB staining of *N. benthamiana* leaf expression with Gh-LYK2-ED, single LysM domain deletion mutants of Gh-LYK2-ED and EV. EV: empty vector. **(C)** Phenotype of *N. benthamiana* leaf expression with Gh-LYK2-ED, single LysM domain deletion mutants of Gh-LYK2-ED and EV. **(D)** Trypan blue staining of the same leaf presented in **(C)**.

A recent study reported that the ED and TM of AtCERK1 in an *A. thaliana* mutant (*cerk1-4*) could deregulate cell death independently of chitin signaling (Petutschnig et al., 2014). And the reactive oxygen species (ROS) is a signal in plant cell death (Van Breusegem and Dat, 2006). We next asked whether the cotton LYKs could induce a burst of ROS. When the Gh-LYKs and their truncated mutants were transiently expressed in *N. benthamiana*, we could not detect accumulation of ROS as determined by DAB staining in Gh-LYK2- (Figure S9A), Gh-LYK2-ID- (Figure 7A), Gh-LYK1-ED- (Figure S9B), and empty vector (EV)-infiltrated leaves, while this accumulation was detectable in Gh-LYK2-ED-infiltrated leaves (Figure 7A). As mentioned above, all three LysMs are important for Gh-LYK2 chitin-binding ability. To figure out whether the process of ROS burst induced by Gh-LYK2-ED is dependent on its chitin-binding activity, three single domain-deleted mutants of Gh-LYK2 were transiently expressed in tobacco leaves. The DAB staining results show that all mutants could induce ROS accumulation (Figure 7B), while the transfected leaves without DAB treatment showed no significant staining (Figure S9C). We also tested whether the ROS accumulation was associated with programed cell death (PCD). In the infiltrated areas, water soaking symptoms appeared 3 days post-infiltration when Gh-LYK2-ED or LysM deletion mutants were expressed (Figure 7C). Additionally, uneven blue spots in the infiltrated areas could be visualized upon trypan blue staining (Figure 7D). These results suggest that ectopic expression of Gh-LYK2-ED results in ROS generation and chitin-independent induction of cell-death.

## Discussion

In recent years, the function of LysM receptors in plant innate immunity has been investigated in model plants such as *Arabidopsis* and rice (Buist et al., 2008; Gust et al., 2012; Tanaka et al., 2013; Shinya et al., 2015). In *Arabidopsis*, LysM-containing proteins, including CERK1, LYK4, and LYK5, act as essential chitin PRRs that regulate the chitin signaling pathway (Miya et al., 2007; Wan et al., 2012; Cao et al., 2014). AtLYK3 is reported to be responsible for the negative regulation of immunity (Liang et al., 2013; Paparella et al., 2014). However, the function of At-LYK2 in defense remains unclear, as a single *atlyk2* mutant or a triple *atlyk2*/*atlyk3*/*atlyk5* mutant display no significant changes in the expression of defense-related marker genes in response to chitin treatment (Wan et al., 2008, 2012). In this study, we found that both Gh-LYK1 and Gh-LYK2 are important for *Verticillium* wilt resistance in cotton, because silencing of these two genes compromised resistance to both defoliating and non-defoliating *V. dahliae* isolates. We also simultaneously knocked down the expression of *Gh-LYK1* and *Gh-LYK2* with a CLCrV VIGS vector. The double-silenced plants developed much more severe wilting symptoms compared to the single-silenced or control plants, suggesting that two members of the LYK family, Gh-LYK1 and Gh-LYK2, act additively to promote *Verticillium* wilt resistance in cotton. Recent studies have demonstrated that LysM proteins play roles in sensing chitin of fungal origin or PGN of bacterial origin to mediate plant basal immunity (Willmann et al., 2011; Ao et al., 2014; Kouzai et al., 2014; Mesnage et al., 2014). Based on our observations, the expression of *Gh-LYK1* and *Gh-LYK2* increased during either chitin or PGN treatment, and two plant downstream defense genes, namely *WRKY53* and *MPK3*, were down-regulated in *Gh-LYK1* or *Gh-LYK2* knockdown plants after PAMP treatment. It is tempting to speculate that, like CERK1, Gh-LYK1, and Gh-LYK2 could play roles in both resistance to fungal and bacterial infection.

Although the molecular mechanism of chitin recognition by the LysM receptors in rice and *Arabidopsis* has been gradually revealed in recent years (reviewed in Böhm et al., 2014; Zipfel, 2014; Shinya et al., 2015), the function of LYK proteins in non-model plants remains obscure. Our chitin-binding assays showed that the ED of Gh-LYK1 and Gh-LYK2 could directly bind chitin, and that all three LysMs are required for their chitin-binding activity. The lack of kinase activity of Gh-LYK2 implies that it may interact directly with some other factors, rather than Gh-LYK1, to form a receptor complex involved in chitin perception.

The homodimerization of LysM-containing proteins has been described in plant PRRs such as AtCERK1, At-LYK5, and Os-CEBiP, as well as in the fungal effector Ecp6 (Liu et al., 2012; Sánchez-Vallet et al., 2013; Cao et al., 2014; Hayafune et al., 2014). These findings suggest that formation of homo-complexes in LysM-containing proteins is important for chitin-induced signaling. Based on our observations, both the ED of Gh-LYK2 and the full length protein can form homodimers in the absence of the chitin (Figures 5A,B and Figure S6A). And the homodimer formation does not depend on disulfide bonds, because extra treatment with DTT would not eliminate the homodimer bands, as shown in the immunoblot assay (Figures 5A,B). These data suggest that the homodimer form of Gh-LYK2 could exist in the cell in the abscence of chitin.

Recent work in *Arabidopsis* proved that the ED and TM of *cerk1-4* were sufficient to deregulate cell death (Petutschnig et al., 2014). We found that ectopic expression of the ED of Gh-LYK2, but not Gh-LYK2-ID or Gh-LYK1-ED (Figure S9B), can induce ROS burst and PCD (Figure 7A). More interestingly, ectopic expression of LysM deletion mutants was sufficient to induce ROS accumulation in *N. benthamiana* (Figure 7B). This suggests that the LysM-related cell death regulation might be chitin-independent, because any single LysM deletion mutation led to a loss of the chitin-binding ability of Gh-LYK2 (Figure 4D). During the arms race between plants and pathogens, fungal and bacterial pathogens have evolved some effector molecules to interact with host receptors and subvert host immunity (Rosebrock et al., 2007; Shan et al., 2008; Xiang et al., 2008; Dou and Zhou, 2012; Macho and Zipfel, 2015). The best-characterized LYK in *Arabidopsis*, CERK1, has been reported to be targeted by AvrPtoB for degradation (Gimenez-Ibanez et al., 2009). In our study, the ED or the partial ED (LysM deletion mutants), rather than its full-length form, of Gh-LYK2 could induce ROS burst in plants without pathogen challenge (Figures 7A,B), and the expression of *Gh-LYK2* was up-regulated during *V. dahliae* infection (Figure 1B). One possible scenario is that, during pathogen infection, Gh-LYK2, as an important receptor, is targeted by some specific effector to be degraded. Concomitantly, the integrity of Gh-LYK2 could be monitored in the plant so that the breakdown products, i.e., ED, might act as signals to activate the downstream PCD and restrict pathogen spread. In order to speculate about which part of Gh-LYK2-ED could induce PCD, we searched the SMART database (Letunic et al., 2015) and found a specific domain, belonging to the actin-crosslinking proteins superfamily, located between the SP and LysM1. Functional annotation indicates that members of this superfamily are involved in the ARP2/3-dependent actin polymerization pathway, which is important for forming autophagosomes (Goley and Welch, 2006; Höhfeld, 2016). In this pathway, filamin dimers and subsequent ubiquitination mediate the membrane isolation and motility of autophagosomes. This may be a possible explanation as to why Gh-LYK2-ED and derivatives show dimeric and smear bands in western blot upon transient expression *in planta* (Figure 4D). Using a specific antibody to differentially detect the ecto- and intracellular domains of Gh-LYK2 under physiological conditions may help to unravel the role of GH-LYK2 ED in cell death control.

In conclusion, we show that two LysM receptor-like kinases (Gh-LYK1 and Gh-LYK2) contribute to resistance against *Verticillium* wilt in cotton. Additionally, Gh-LYK2 might play a role in the regulation of PCD, which needs to be investigated further.

## Author contributions

ZG, TL, BZ, and XZ: planned and designed the research; ZG and TL: performed the experiments; ZG, TL, BD, FL, QW, SQ, FY, TC, YY, JW, GW, BZ, and XZ: analyzed the data; ZG, TL, BD, BZ, and XZ: wrote the manuscript.

### Conflict of interest statement

The authors declare that the research was conducted in the absence of any commercial or financial relationships that could be construed as a potential conflict of interest.

## Supplementary material

Figure S1 Transcriptional analysis of *Gh-LYK5* in cotton plant after *V. dahliae* infection. Relative expression of Gh-LYK5 in roots determined through quantitative reverse transcription PCR (qRT-PCR) at 0, 1, 2, 4, 8, 12 days post *V. dahliae* inoculation. Values with the same lower case letter above the error bar were not significantly different according to Duncan's multiple range tests (*P* < 0.05).

Figure S2 Relative fugal biomass in Gh-LYK5-silenced cotton plants after *V. dahliae* challenge. The *V. dahliae* biomass in the silenced and non-silenced plants challenged with *V. dahliae* was estimated at 30 days post *V. dahliae* inoculation. The error bars represent SE of the biological replicates. Values with the same lower case letter above the error bar were not significantly different according to Duncan's multiple range tests (*P* < 0.05).

Figure S3 Transcriptional analysis of Gh-LYKs, WRKY53, and MPK3 in cotton c.v. 3,503 root after PAMP treatment. **(A)** The relative expressions of Gh-LYKs, WRKY53 and MPK3 after 30 min treatment of flg22 or chitin. **(B)** The relative expressions of Gh-LYKs, WRKY53, and MPK3 after 24 h flg22-treated cotton root.

Figure 4 Phylogenetic analysis of Gh-LYKs with homologies to *A. thaliana*.

Figure S5 The subcellular localization of Gh-LYK1 and Gh-LYK2 after plasmolysis treatment. Confocal microscopy images of tobacco leaf cells infiltrated with *Agrobacterium* harboring the Gh-LYK1-GFP **(A)** and Gh-LYK2-GFP **(B)** fusion constructs before (control panel) or after plasmolysis treatment.

Figure S6 BiFC assays of Gh-LYK1 and Gh-LYK2 in *N. benthamiana* leaves. **(A)** YFP fluorescence (yellow) was observed as a consequence of self-interaction of Gh-LYK2 tagged with 2YN and 2YC, but not in the self-interaction of Gh-LYK1 tagged with 2YN and 2YC or interaction between Gh-LYK1and Gh-LYK2 tagged with 2YN and 2YC. **(B)** The immunoblotting of Gh-LYK1 fused proteins were detected with anti-HA epitope antibody in BiFC assays.

Figure S7 Alignment of Gh-LYK1 and Gh-LYK2 kinase domain. Alignment was performed using ClustalW with default parameters. The red arrows indicated the amino-acid residues missed or changed in the sub-kinase domains.

Figure S8 The split-ubiquitin yeast two hybrid assay of Gh-LYK1 and Gh-LYK 2.

Figure S9 Transient expression of Gh-LYK2 **(A)** or Gh-LYK1-ED **(B)** could not induce the accumulation of ROS in *N. benthamiana* leaf and the EV/GH-LYK2-ED and derivates infiltrated leaf showed no significant differences without DAB staining **(C)**.

Table S1 Primers used in this study.
