# Supplementary figures and images for: Two Lysin-Motif Receptor Kinases, Gh-LYK1 and Gh-LYK2, Contribute to Resistance against Verticillium wilt in Upland Cotton
Source: Front Plant Sci. 2017 Dec 13;8:2133. doi: 10.3389/fpls.2017.02133 (PMC5733346; doi:10.3389/fpls.2017.02133)

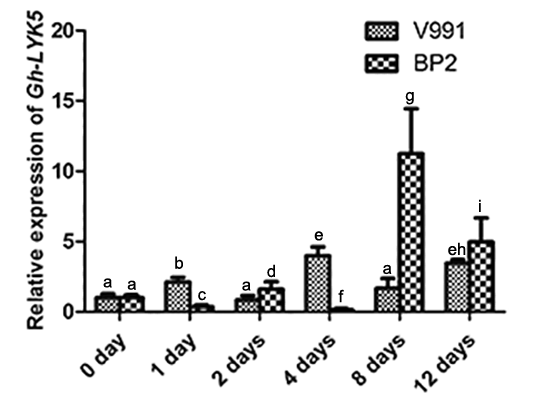

Supplement: Figure S1 — Transcriptional analysis of Gh-LYK5 in cotton plant after V. dahliae infection. Relative expression of Gh-LYK5 in roots determined through quantitative reverse transcription PCR (qRT-PCR) at 0, 1, 2, 4, 8, 12 days post V. dahliae inoculation. Values with the same lower case letter above the error bar were not significantly different according to Duncan's multiple range tests (P < 0.05). [file Image1.TIF]

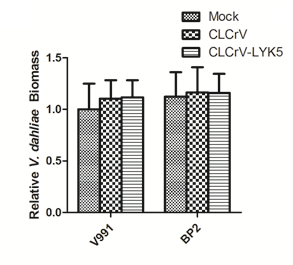

Supplement: Figure S2 — Relative fugal biomass in Gh-LYK5-silenced cotton plants after V. dahliae challenge. The V. dahliae biomass in the silenced and non-silenced plants challenged with V. dahliae was estimated at 30 days post V. dahliae inoculation. The error bars represent SE of the biological replicates. Values with the same lower case letter above the error bar were not significantly different according to Duncan's multiple range tests (P < 0.05). [file Image2.TIF]

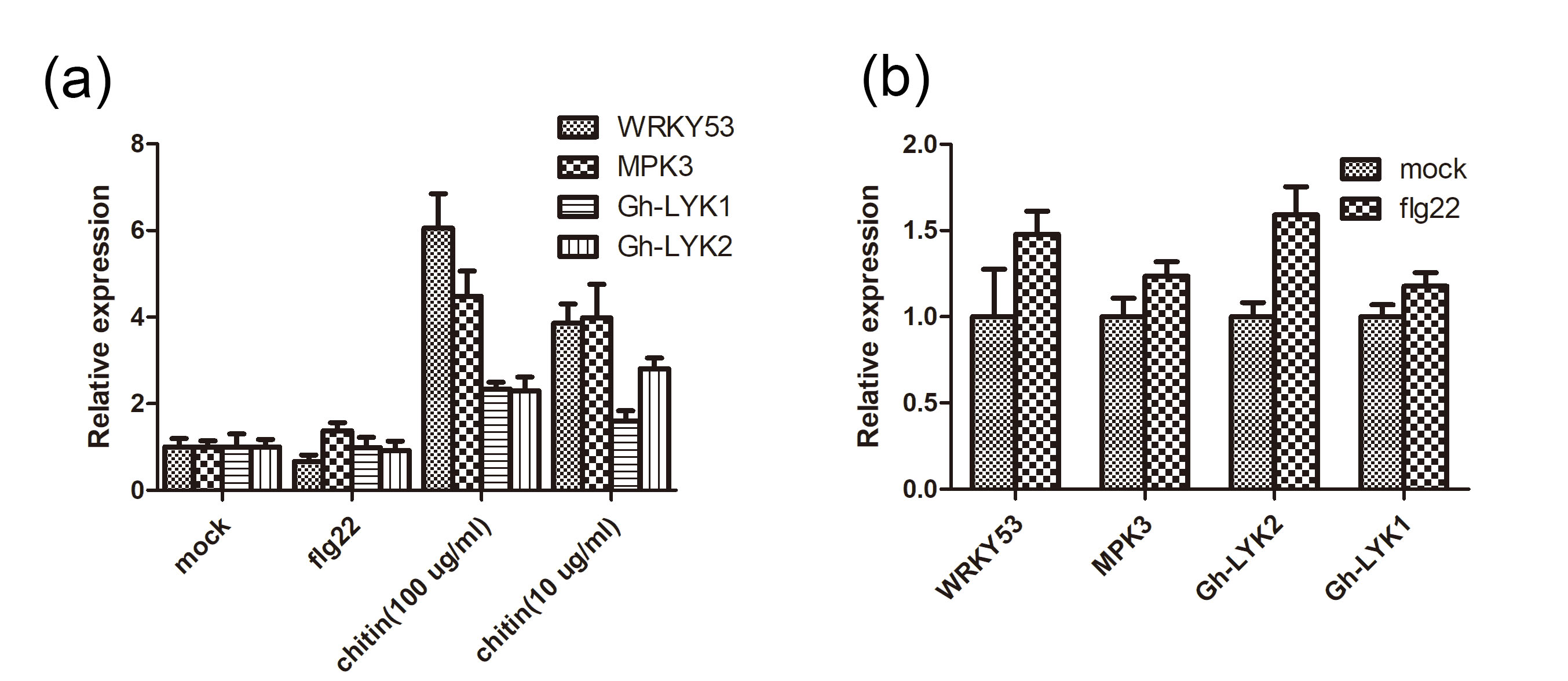

Supplement: Figure S3 — Transcriptional analysis of Gh-LYKs, WRKY53, and MPK3 in cotton c.v. 3,503 root after PAMP treatment. (A) The relative expressions of Gh-LYKs, WRKY53 and MPK3 after 30 min treatment of flg22 or chitin. (B) The relative expressions of Gh-LYKs, WRKY53, and MPK3 after 24 h flg22-treated cotton root. [file Image3.JPEG]

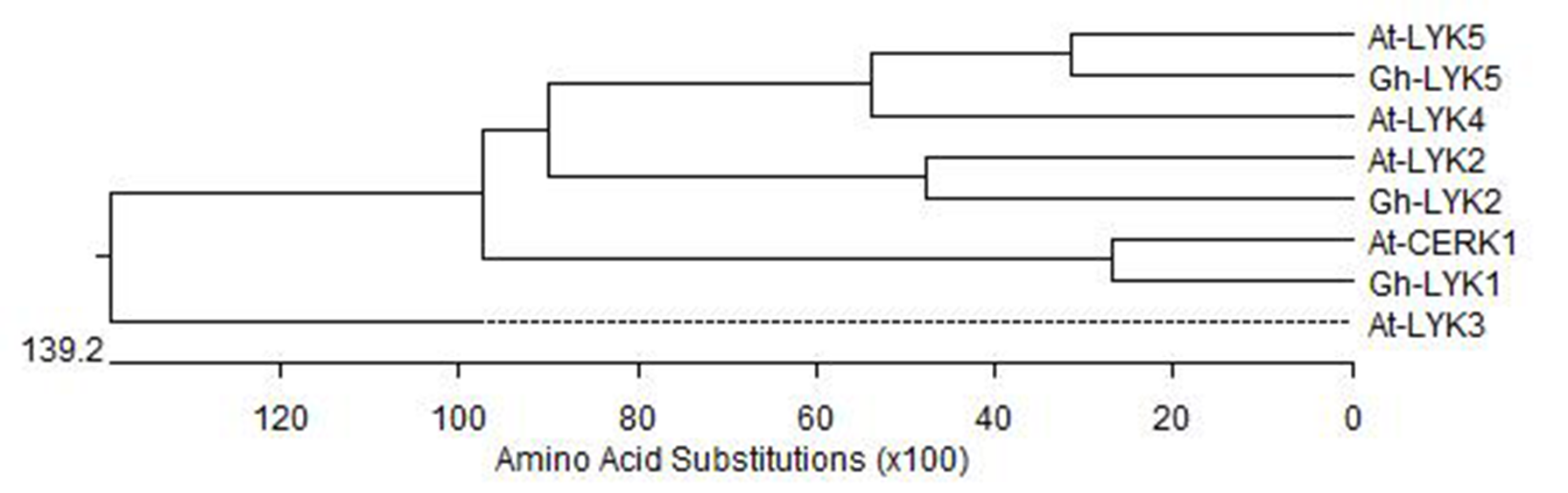

Supplement: Figure 4 — Phylogenetic analysis of Gh-LYKs with homologies to A. thaliana. [file Image4.TIF]

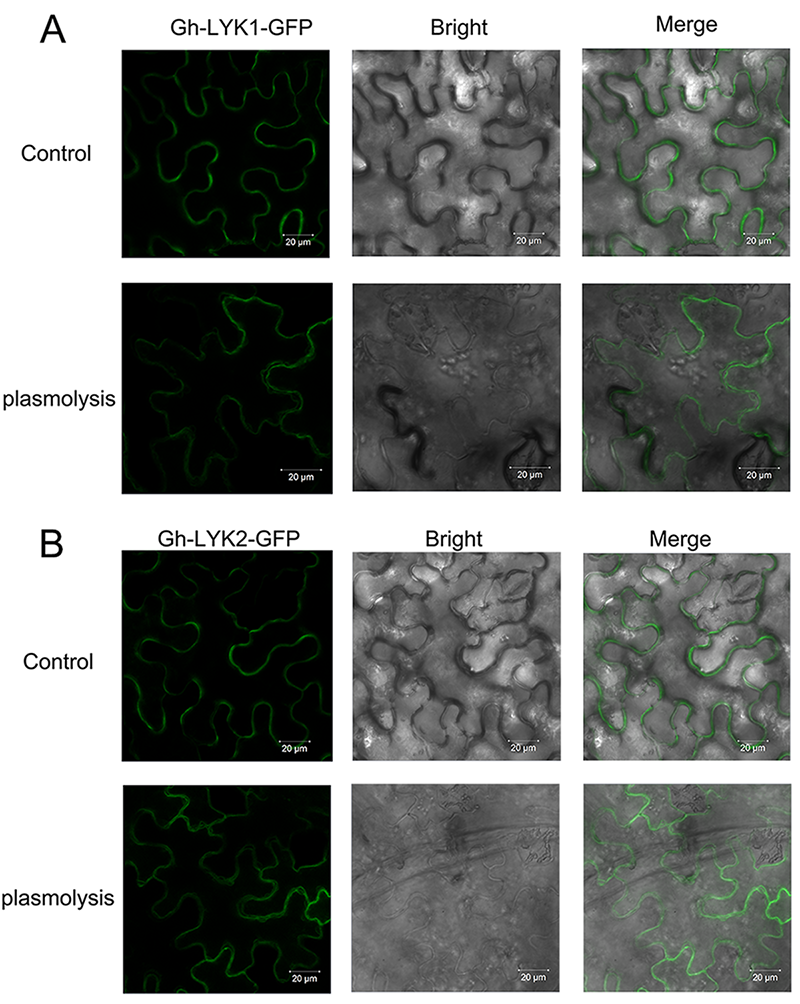

Supplement: Figure S5 — The subcellular localization of Gh-LYK1 and Gh-LYK2 after plasmolysis treatment. Confocal microscopy images of tobacco leaf cells infiltrated with Agrobacterium harboring the Gh-LYK1-GFP (A) and Gh-LYK2-GFP (B) fusion constructs before (control panel) or after plasmolysis treatment. [file Image5.tif]

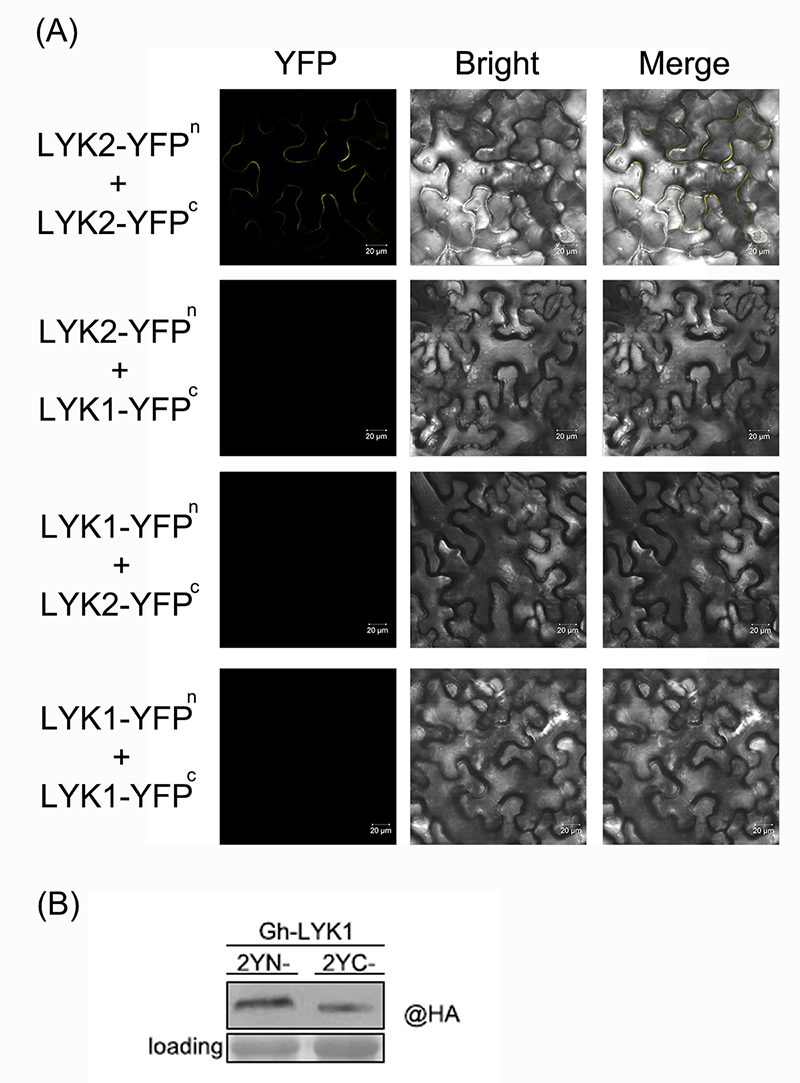

Supplement: Figure S6 — BiFC assays of Gh-LYK1 and Gh-LYK2 in N. benthamiana leaves. (A) YFP fluorescence (yellow) was observed as a consequence of self-interaction of Gh-LYK2 tagged with 2YN and 2YC, but not in the self-interaction of Gh-LYK1 tagged with 2YN and 2YC or interaction between Gh-LYK1and Gh-LYK2 tagged with 2YN and 2YC. (B) The immunoblotting of Gh-LYK1 fused proteins were detected with anti-HA epitope antibody in BiFC assays. [file Image6.TIF]

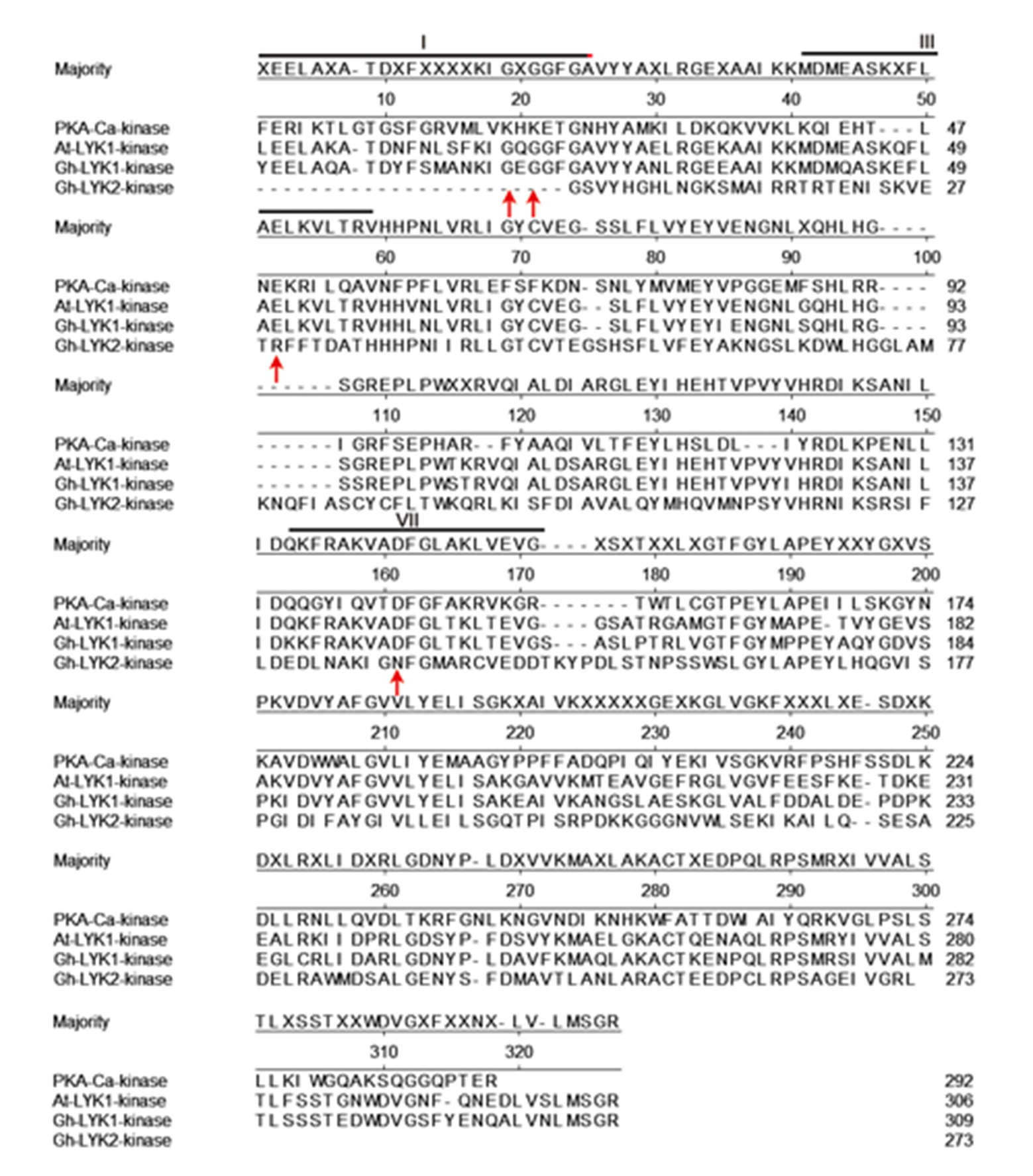

Supplement: Figure S7 — Alignment of Gh-LYK1 and Gh-LYK2 kinase domain. Alignment was performed using ClustalW with default parameters. The red arrows indicated the amino-acid residues missed or changed in the sub-kinase domains. [file Image7.TIF]

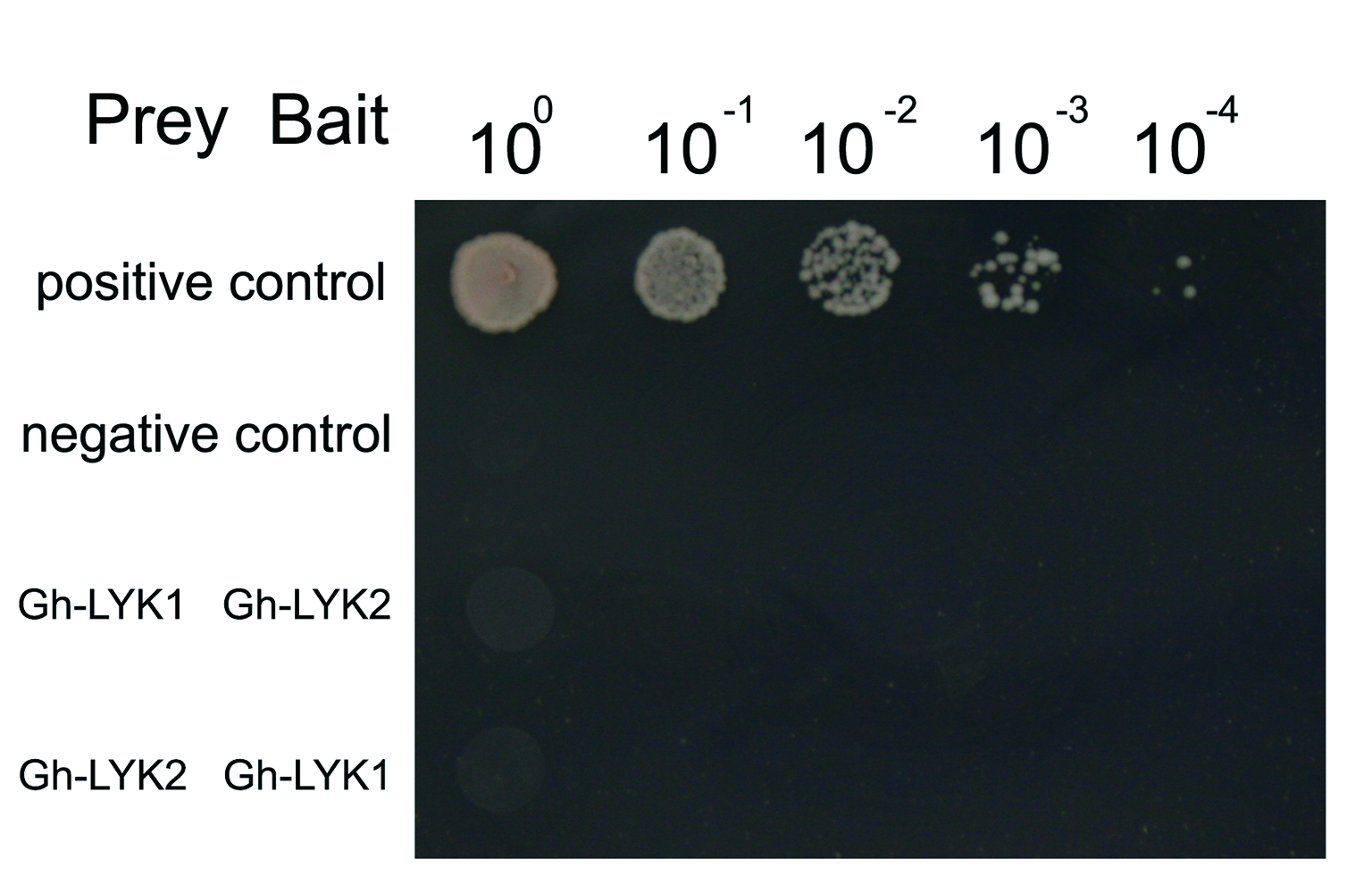

Supplement: Figure S8 — The split-ubiquitin yeast two hybrid assay of Gh-LYK1 and Gh-LYK 2. [file Image8.TIF]

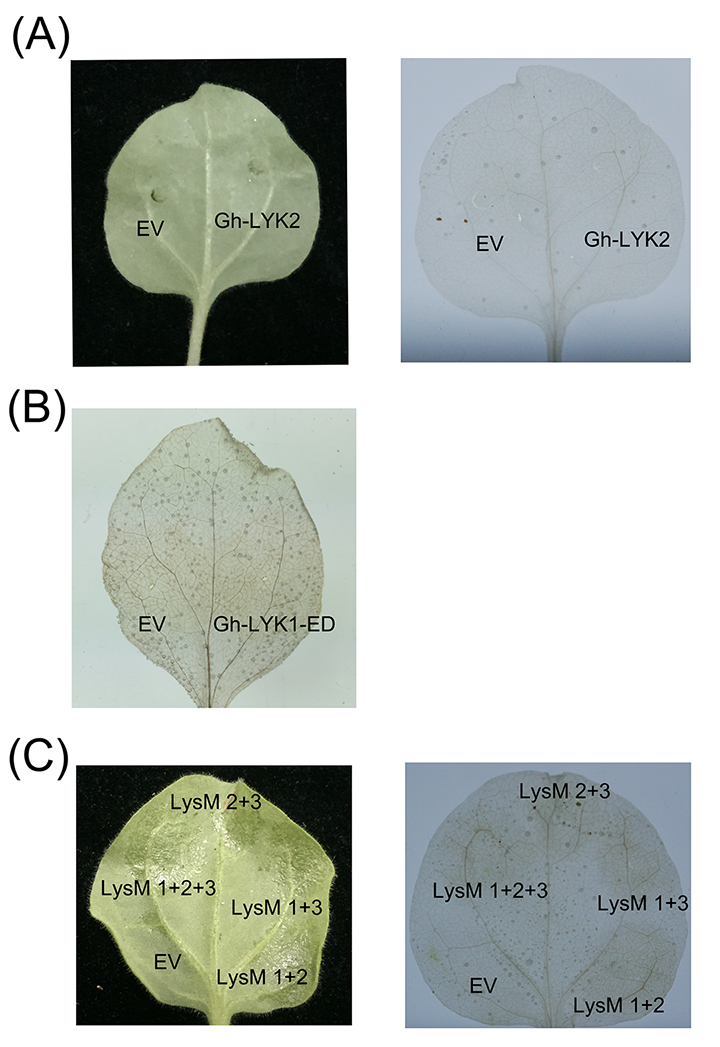

Supplement: Figure S9 — Transient expression of Gh-LYK2 (A) or Gh-LYK1-ED (B) could not induce the accumulation of ROS in N. benthamiana leaf and the EV/GH-LYK2-ED and derivates infiltrated leaf showed no significant differences without DAB staining (C). [file Image9.TIF]
