# Supplementary material for: Two Lysin-Motif Receptor Kinases, Gh-LYK1 and Gh-LYK2, Contribute to Resistance against Verticillium wilt in Upland Cotton
Source: Front Plant Sci. 2017 Dec 13;8:2133. doi: 10.3389/fpls.2017.02133 (PMC5733346; doi:10.3389/fpls.2017.02133)
Supplement: Table S1 — Primers used in this study. [file Table1.DOC]

| **Name** | **Sequence 5’-3’*** | **Brief Description** |
| --- | --- | --- |
| **For plasmid construct** | | |
| CLCrV-F | AAGCTTACCTGAACTTCCAAGTCTGGA | For CLCrV detection |
| CLCrV-R | GCCTAATGGGTATAGAGCAAAATGGCA |
| D_cotton_018813 F: | gactagtcATGTTACTTAAATTATTCCC | For Gh-LYKs detection |
| D_cotton_018813 R: | CCttaattaaGGCTATCGTCCAGACATAAGGTTGAC |
| D_cotton_000932 F: | gactagtcATGCTGAAATTAATCTCGATTC |
| D_cotton_000932 R: | CCttaattaaGGTTATCTTCCGGACATAAGGTTGACG |
| LYK4-17682F: | ATGGAGATGAAAGTGAACAGTTTTTCTG |
| LYK4-17682R: | TCAAGAGCTCCCAGTAGATGTTTG |
| D_cotton_017683 F: | gactagtcATGGATTCTTTGTCTCTTATTTCTC |
| D_cotton_017683 R: | CCttaattaaGGTCATGAATCCAACGCCGATGAAGC |
| D_cotton_035752F: | gactagtcATGCATCTGTAATGAGTAAAATATAC |
| D_cotton_035752R: | CCttaattaaAGGTCACAGATTGGATGTTGTGGATGATG |
| D_cotton_019720F : | gactagtcATGGCAGTTGGAAGATTGAAACC |
| D_cotton_019720R: | CCttaattaaGGCTACCTCGCACTGGTTAGAGATGTGG |
| D_cotton_014206F: | gactagtcATGCTCATAACCATGGTTTCAC |
| D_cotton_014206R: | CCttaattaaTCATCTTCCATTGAAAACTCCACTG |
| LYK1 full F | ATGCTGAAATTAATCTCGATTC | For full length Gh-LYK1 clone |
| LYK1 full R | TTATCTTCCGGACATAAGGTTGACG |
| LYK2 full F | ATGCATCTGTAATGAGTAAAATATAC | For full length Gh-LYK2 clone |
| LYK2 full R | TCACAGATTGGATGTTGTGGATGATG |
| LYK5 full F | gactagtcATGGCAGTTGGAAGATTGAAACC | For full length of Gh-LYK5 clone |
| LYK5 full R | CCttaattaaGGCTACCTCGCACTGGTTAGAGATGTGG |
| LYK1 F *Spe*I | GactagtCAACTGTTGCTGCTTCTCCCG | For Gh-LYK1 VIGS |
| LYK1 R *Pac*I | CCttaattaaGGGGCTTAGATTGCCATTCTCG |
| LYK2 F *Spe*I | gactagtcCTCATCACCACCCAAACATAA | For Gh-LYK2 VIGS |
| LYK2 R *Avr*II | CCcctaggGGTTCAACACACCTTGCCATACC |
| LYK5 F *Spe*I | gactagtcGAGACTTTTGGTGCTGATGAG | For Gh-LYK5 VIGS |
| LYK5 R *Avr*II | cccctaggggGTAACGAGTTAAGGATTCGAT |
| LYK2 silencing F *Pac*I | CCttaattaaGGCATCACCACCCAAACATAAT | For GhLYK1 and Gh-LYK2 double VIGS |
| LYK2 silencing R *Asc*I | AggcgcgccTCTTTGCATTCAAATCTTCAT |
| LYK1 ED F *Bam*HI | ggatccATGAGGTGCAGCAGAGGCTGTGA | For Gh-LYK1 ectodomain clone |
| LYK1 ED R *Xho*I | CCGctcgagCGGTCATGAAATTCCTTTGCTTGATTTGAGGGG |
| LYK2 F ED *Bam*HI | ggatccATGGCATCTGTAATGAGTAAAATATACTTG | For Gh-LYK2 ectodomain clone |
| LYK2 R ED *Xho*I | CCGctcgagCGGTCATCCCACTTTCCACATCCTTGGC |
| LYK1 ID F *Bam*HI | ggatccGCTAAATTTTTCCGAAAGAAGTC | For Gh-LYK1 intracellular domain clone |
| LYK1 ID R *Xho*I | ctcgagTTATCTTCCGGACATAAGGTTGACGAGAGC |
| LYK2 ID F *Bam*HI | ggatccCGATTGAAGAGGAAAAAGAAGAAGC | For Gh-LYK2 intracellular domain clone |
| LYK2 R ID *Xho*I | ctcgagTCACAGATTGGATGTTGTGGATGATG |
| LYK1 F *Sal*I | ATgtcgacATGCTGAAATTAATCTCGATTCTGTTGCTATTAATC | For Gh-LYK1 subcellular localization |
| LYK1 R *Bam*HI | ggatccTTATCTTCCGGACATAAGGTTGACGAGAGC |
| LYK2 F *Sal*I | ATgtcgacATGGCATCTGTAATGAGTAAAATATACTTGATAGC | For Gh-LYK2 and Gh-LYK2-ZN905 subcellular localization |
| LYK2 R *Bam*HI | ggatccTCACAGATTGGATGTTGTGGATGATG |
| Bt3-LYK2 full F NotI | gcggccgcATGGCATCTGTAATGAGTAAAATATA | For DUALmembrane system clone |
| Bt3-LYK2 R *Spe*I mstop | actagtCAGATTGGATGTTGTGGATGATG |
| PR3-LYK2 F full SpeI | actagtATGGCATCTGTAATGAGTAAAATATA |
| PR3-LYK2 *Eco*RI R mstop | gaattcCAGATTGGATGTTGTGGATGATG |
| Bt3-LYK1 F *Pst*I | ctgcagATGCTGAAATTAATCTCGATTCTGTT |
| Bt3-LYK1*Spe*I R mstop | actagtTCTTCCGGACATAAGGTTGACG |
| PR3-LYk1 full F *Spe*I | actagtATGCTGAAATTAATCTCGATTCTGTT |
| PR3-LYK1 R *Nco*I mstop | ccatggTCTTCCGGACATAAGGTTGACG |
| 2-LysM3-del R *Xba*I | aatctagaAAGTAAGAGCCTTACTTTCAAAGCG | For Gh-LYK2 LysM deletion mutant clone |
| 2-LysM1 F *Bam*HI | ggatccATGAGGTGCAGCAGAGGCTGTGA |  |
| 2-LysM2-del OV1 R | GGTACCACTAACCGGACTTTGTCACCTGTTAAATCAGCATGG |  |
| 2-LysM2-del OV2 F | CCATGCTGATTTAACA*GGTGACAAAGTCCGGTTAGTGGTACC* |  |
| 2-LysM1-del OV1 R | CCATTGTTGCATTTACAATCAATATAAAAGCTCAGATTGGAAAGAG |  |
| 2-LysM1-del OV1 F | CTCTTTCCAATCTGAGCTTTTAT*ATTGATTGTAAATGCAACAATGG* |  |
| 1-LysM3-del R *Xba*I | aatctagaCGTAACAAACAAACCGTAATCCTTGG | For Gh-LYK1 LysM deletion mutant clone |
| 1-LysM2-del OV1R | CCACCCCACTATCGGGGGTGAAAACATGGCCAAGGAAATCG |  |
| 1-LysM2-del OV2 F | CGATTTCCTTGGCCATGTTTTCACC*CCCGATAGTGGGGTGG* |  |
| 1-LysM1-del OV1 R | GCAATCACAAGGGAATGCTAAAGCCAAGTCACAGCC |  |
| 1-LysM1-del OV2 F | GGCTGTGACTTGGCTTTAGCA*TTCCCTTGTGATTGC* |  |
| **For qPCR analysis** | | |
| JAGN889 | GTCCACAACAGAGGATTGGGATGT | For qPCR analysis of *Gh-LYK1* |
| JAGN890 | CTGTTCGCTGATCTATCGTCCAGA |
| JAGN881 | ATTGGGAACTTTGGTATGGCAAG | For qPCR analysis of *Gh-LYK2* |
| JAGN882 | CATAAGCAAATATGTCAATGCCTG |
| JAGN885 | TTCTGGACAAACTCATAAACCAAG | For qPCR analysis of *Gh-LYK3* |
| JAGN886 | ATGTGCATAGGGATATCAAGACAA |
| JAGN879 | GGTTATATGGCTCCTGAGTACATTG | For qPCR analysis of *Gh-LYK4* |
| JAGN880 | CCTTCAAGCACCACTCTGATAGAT |
| JAGN879 | GGTTATATGGCTCCTGAGTACATTG | For qPCR analysis of *Gh-LYK5* |
| JAGN880 | CCTTCAAGCACCACTCTGATAGAT |
| JAGN1161 | GTTGGGACTCTTGGTGGTAGACG | For qPCR analysis of *WRKY53* |
| JAGN1162 | AATTCTGCTAATTTTCCTCCATGTC |
| JAGN1163 | AGGACATGATTTACCAGGAGGCC | For qPCR analysis of *MPK3* |
| JAGN1164 | AAGTGGACTGACCTCGTGAAAGC |
| UBQ14-qF | CAACGCTCCATCTTGTCCTT | For qPCR analysis of *UBQ14* |
| UBQ14-qR | TGATCGTCTTTCCCGTAAGC |

*Restriction sites are shown in lower case.
